# Renal function change after switching tenofovir disoproxil fumarate for tenofovir alafenamide in the HIV-positive patients of a metropolitan sexual health service

**DOI:** 10.1186/s12981-019-0256-9

**Published:** 2019-12-07

**Authors:** Dane Turner, Douglas Drak, Catherine C. O’Connor, David J. Templeton, David M. Gracey

**Affiliations:** 1 Sydney Medical School, University of Sydney, Sydney, NSW Australia; 2 Central Clinical School, University of Sydney, Sydney, NSW Australia; 3 RPA Sexual Health, Sydney Local Health District, Sydney, NSW Australia; 4 Kirby Institute, UNSW Sydney, Sydney, NSW Australia; 5 Renal Unit, Royal Prince Alfred Hospital, Sydney Local Health District, Sydney, NSW Australia

**Keywords:** Tenofovir disoproxil fumarate, Tenofovir alafenamide, HIV, Renal function

## Abstract

**Background:**

Tenofovir disoproxil fumarate (TDF) is widely used in the management of HIV-infection, but has been associated with renal impairment in a small proportion of patients. Tenofovir alafenamide (TAF), a novel prodrug of tenofovir, causes less renal impairment and can improve renal function in patients switched from TDF. The factors which predict improved renal function in patients switching from TDF to TAF have yet to be described.

**Aim:**

To determine which patient factors are associated with an improvement in renal function following the switch from a TDF- to a TAF-based HIV antiretroviral regimen.

**Methods:**

A retrospective analysis was performed of a cohort from a publicly funded sexual health clinic in Sydney, Australia. All HIV-positive clinic patients switched from a TDF- to TAF-containing regimen between January 2016 and August 2018 were eligible for inclusion. Laboratory results were obtained from patients’ electronic medical records. The statistical significance of differences between pre- and post-switch means was determined by paired t-tests, adjusted for baseline values, and associations between continuous variables by univariate linear regression.

**Results:**

79 patients met inclusion criteria. The majority were male (89%), with a median age of 44 years (IQR: 34.5 to 53). Patients had a mean pre-switch estimated glomerular filtration rate (eGFR) of 95 ± 2 mL/min/1.73 m^2^, and there was no significant change post-switch (p = 0.062). Pre-switch eGFR was a significant predictor of the magnitude of eGFR change after the switch (p < 0.001), but there was no significant association with age (p = 0.189), cumulative TDF exposure (p = 0.454) or baseline urinary protein to creatinine ratio (p = 0.814).

**Conclusion:**

While there was no significant difference in mean eGFR, in patients switched from TDF to TAF, baseline eGFR was a significant predictor of the change in eGFR. This suggests that patients on TDF with poorer baseline renal function would benefit more from switching to TAF. Further study to explore this association is warranted.

## Background

Modern combination antiretroviral therapy (cART) is highly efficacious in virological suppression of HIV-infection [1]. Novel drug development is ongoing however, to improve the tolerability and safety of cART regimens, particularly given the need for lifelong therapy. Tenofovir alafenamide (TAF) is a recent product of this initiative and has been shown to be similarly efficacious to the older prodrug tenofovir disoproxil fumarate (TDF) with a lower incidence of adverse renal events [2].

TDF remains widely used and a recommended first-line antiretroviral by major international societies [1, 3]. It is, however, associated with proximal tubular dysfunction including impaired estimated glomerular filtration rate (eGFR), proteinuria and, rarely, Fanconi syndrome [4]. These effects are largely reversible with the discontinuation of TDF [5, 6] and recent evidence has emerged that these adverse effects can also be ameliorated by switching to TAF [7–10]. Not all patients benefit equally from the switch from TDF to TAF. One large study of patients with impaired creatinine clearance (30 to 69 mL/min) and multiple comorbidities showed no improvement in eGFR from switching [11].

There are limited other data on factors which may predict improvements in renal function with the switch of antiretroviral regimen. There was no apparent difference in the response of patients with or without pre-existing renal impairment (eGFR < 90 mL/min) in the data supplement of one of the larger switch studies, but no statistical analysis was performed [7]. Our aim was therefore to address this paucity of data and investigate factors, in a clinical setting, which predict improvement in renal function among HIV-positive patients switched from TDF to TAF-containing cART.

## Methods

This study was conducted in accordance with the Declaration of Helsinki and applicable local and national guidelines. Ethics approval for this project was obtained from the RPAH Zone Human Research Ethics Committee of the Sydney Local Health District. This included a waiver of consent, permitting access to identified health data.

A retrospective review was conducted of HIV-positive patients who attend an inner-city publicly funded sexual health service in Sydney, Australia for routine HIV care. Study inclusion criteria were: switching from a TDF- to TAF-containing regimen between January 2016 and August 2018 (inclusive), aged ≥ 18 years at time of switch and at least one eGFR measurement within the 6 months prior to the switch date and at least one eGFR measurement within 9 months after the switch date. There were no specific exclusion criteria.

HIV medication history and laboratory results were extracted from the electronic medical records of eligible patients. Laboratory results collected were serum creatinine, urinary protein to creatinine ratio (uPCR) and serum phosphate, with eGFR calculated using the CKD-EPI equation [12]. Clinic visits occurred on approximately a 3- to 6-monthly basis and all results were therefore attributed to the nearest 3-month period as measured from the “switch date”, defined as the date patients were provided with their first prescription for a TAF-containing regimen. The “pre-switch” measurement was defined as the laboratory result at the switch date or, if unavailable, that immediately preceding it. The “post-switch” measurement was defined as the first laboratory result after at least 3 months post-switch. Both pre- and post-switch measurements were limited to a 6-month window. Where there were multiple measures in a testing period, the mean of test results was calculated and used for the analysis.

Statistical analyses were preformed using SPSS v20 (IBM, New York, USA) and figures were generated using Prism v7 (GraphPad Software, California, USA). Comparisons between pre- and post-switch laboratory measures were performed by using paired t-tests with baseline values as a covariate, to adjust for regression to the mean. This procedure increases precision by adjusting for the mean-centered independent variable [13], in our study, the baseline value. Linear regression was used to assess the association of covariates on the change in eGFR post-switch and those with a p-value of < 0.1 in univariate analysis were considered in the multivariate model. The covariates analysed in univariate analysis were age, cumulative TDF exposure, pre-switch eGFR and pre-switch uPCR. Data are presented as either mean ± SEM or median (IQR). For all analyses, p < 0.05 was considered statistically significant.

## Results

A total of 79 HIV-positive patients were identified that had switched from a TDF- to TAF-containing antiretroviral regimen and were included in the analysis. Almost 90% of patients were male, with a median age of 44 years (IQR 34–53). Patients had been on a TDF-containing regimen for a median of 3.5 (IQR 1.5–4.75) years, with three patients having been on TDF for 3 months or less (Table 1).

**Table 1:** Patient demographics and antiretroviral regimens

Characteristic	n (%) or median (IQR)
Sex	
Male	70 (89)
Female	9 (11)
Age (years)	44 (34.5 to 53)
Cumulative TDF exposure (years)	3.5 (1.5 to 4.75)
Pre-switch TDF regimen	
TDF/FTC/^†^	38 (48)
TDF/FTC/RPV	18 (23)
TDF/FTC/EFV	15 (19)
TDF/FTC/E/COBI	8 (10)
Post-switch TAF regimen	
TAF/FTC/^†^	57 (72)
TAF/FTC/RPV	12 (15)
TAF/FTC/E/COBI	10 (13)

*COBI* cobicistat, *E* elvitegravir, *EFV* efavirenz, *eGFR* estimated glomerular filtration rate, *FTC* emtricitabine, *RPV* rilpivirine, *TAF* tenofovir alafenamide, *TDF* tenofovir disoproxil fumarate

^†^These regimens contained a third agent not otherwise listed in the table. In over three quarters of patients, both pre- and post-switch, the third agent was dolutegravir

Both TDF and TAF were taken by all patients in fixed-dose combination tablets, outlined in Table 1. Those patients listed as receiving TDF/FTC/^†^ or TAF/FTC/^†^ were also on a third agent, not otherwise listed in the table. In over three quarters of patients, both pre and post-switch, this third agent was dolutegravir.

### Renal function pre- and post-switch

All pre- and post-switch measurements occurred 3 to 12 months apart. Prior to switching from their TDF-containing regimens, patients had an eGFR of 95 ± 2 mL/min/1.73 m^2^, a uPCR of 10.0 ± 0.9 mg/mmol and a serum phosphate of 1.05 ± 0.03 mmol/L. Over one-third of patients (38%, 95% CI 27 to 49) had an eGFR < 90 mL/min/1.73 m^2^. Proteinuria (uPCR > 20 mg/mmol) and hypophosphatemia (serum phosphate < 0.81 mmol/L) were present in 4 patients (5%, 95% CI 0 to 12%) each, with no instances of patients with both.

**Fig. 1:**
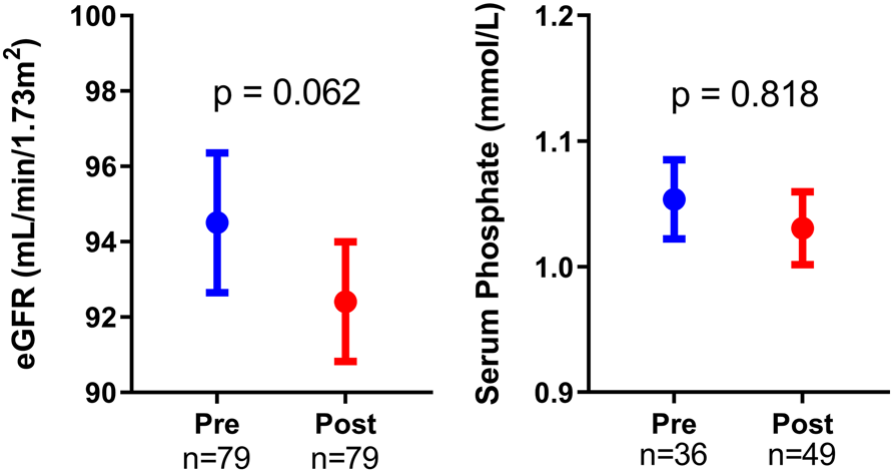
Measures of renal function in patients before switching from tenofovir disoproxil fumarate (Pre) and after switching to tenofovir alafenamide (Post). Graphs show mean and SEM. *eGFR* estimated glomerular filtration rate

Switching to a TAF containing regimen did not lead to significant change in eGFR (mean difference − 2.1 mL/min/1.73 m^2^, 95% CI − 4.3 to 0.1, p = 0.062) or serum phosphate levels (mean difference − 0.01 mmol/L, 95% CI − 0.01 to 0.07, p = 0.818) (Fig. 1). Only eight patients had a post-switch uPCR measurement, limiting statistical comparison. The proportions of patients with an eGFR < 90 mL/min/1.73 m^2^ (41%, 95% CI 34 to 56%) and hypophosphatemia (10%, 95% CI 2 to 19) were also similar to pre-switch measures. Although no patients were found to have proteinuria post-switch, uPCR measurements were only available for eight patients.

### Predictors of eGFR change

In a univariate linear model, there was a significant association between pre-switch eGFR and post-switch change in eGFR (p < 0.001). There was, however, no significant association between the post-switch change in eGFR and age (p = 0.189), cumulative TDF exposure (p = 0.454) and pre-switch uPCR (p = 0.814) (Table 2). As only a single tested predictor had a p < 0.1, no multivariate analysis was performed.

**Table 2:** Univariate regression of potential predictors of the change in eGFR in patients switched from TDF to TAF

Variable	Coefficient (95% CI)	p value
Age	0.120 (− 0.080 to 0.396)	0.189
Cumulative TDF exposure	0.025 (− 0.069 to 0.031)	0.454
Pre-switch eGFR	− 0.381 (− 0.516 to − 0.247)	< *0.001*
Pre-switch uPCR	0.085 (− 0.194 to 0.153)	0.814

Statistically significant p value is in italic

*eGFR* estimated glomerular filtration rate, *TAF* tenofovir alafenamide, *TDF* tenofovir disoproxil fumarate

## Discussion

As far as we are aware, this is the first study to explore potential factors which may predict eGFR response in HIV-positive patients switched from TDF to TAF in a real-world setting. We found that, while there was no overall change in eGFR, patients with lower baseline eGFR have a greater improvement in eGFR post-switch.

Of the large randomized controlled studies which have reported on the renal function in patients switching from TDF to TAF-containing regimens, only Gallant et al. [7] provided data regarding a differential response in subgroups of the participants. In contrast to our findings, participants with a baseline eGFR ≥ 90 and those < 90 mL/min had a similar eGFR response to switching from TDF to TAF. These same subgroups though had a substantial difference in uPCR. Patients with eGFR ≥ 90 who remained on TDF showed an increase in uPCR of 5%, whereas those switched to TAF had a decrease of 11%. In contrast, patients with a baseline eGFR < 90 had a uPCR increase of 16% on TDF and those on TAF a decrease of 20%. Due to only 3 of our patient group having both pre- and post-switch uPCR results, we were unable to assess this change in our study.

Across the major switch studies, switching from TDF to TAF has had a very robust impact on uPCR and a comparatively modest effect on eGFR [7–10]. This difference in effect size may account for the apparent disparity between the eGFR and uPCR responses, by baseline eGFR subgroup, reported by Gallant et al. [7]. Furthermore, as renal function was a secondary endpoint in that study and no detailed subgroup analysis was performed, the impact of confounding factors cannot be excluded. As such, our finding of a predictive role of baseline eGFR in determining eGFR response to switching TDF to TAF does not necessarily conflict with the larger Gallant et al. [7] study.

The majority of patients in our study cohort were on a regimen containing one of dolutegravir, cobicistat or rilpivirine, all of which have been shown to inhibit creatinine secretion from the proximal renal tubule, without affecting glomerular filtration [14]. While our sample size did not permit an exploration of the effect of other agents in the cART regimens, this has been demonstrated in other switch-studies. Mills et al. [8], for example, showed that patients switched from TDF/emtricitabine/efavirenz to a TAF regimen which included cobicistat experienced an increase in serum creatine. These effects are particularly important to consider in real-world clinical settings, where there is likely to be significantly more heterogeneity in cART regimens than clinical trials. It may also account for the trend towards a lower eGFR in post-switch patients in our study, which approached but did not meet statistical significance.

Duration of TDF exposure was not found to predict eGFR change in univariate analysis. Although ongoing TDF exposure has previously been shown to reduce eGFR, the effect is modest [15]. No significant increase in adverse renal events has been observed in patients exposed to TDF for up to 6 years [16]. Given that the majority of our patients had cumulative TDF exposure under 5 years, the lack of association between cumulative TDF exposure and the eGFR response to switching is unsurprising.

Our findings support the recommendation of the European society guidelines, that patients receiving TDF with an eGFR > 60 mL/min should be considered for switch if their eGFR has declined by 5 mL/year for three or more years and/or their eGFR has decreased > 25% from baseline [1]. This advice comes from expert opinion, pending clinical data. While we recognize the potential for regression to the mean to have influenced our analyses, our observational data suggest that such patients would experience a significant improvement in their eGFR when switched to TAF. It is not reasonable, however, to extrapolate this trend to patients with significant renal impairment (eGFR ≤ 60 mL/min/1.73 m^2^). No such patients were included in this study, as they would not have been prescribed TDF due to renal concerns. A single study, conducted in this group, found no improvement in eGFR upon switching from TDF to TAF. Of note, participants in this cohort study had a high burden of comorbidities contributing to renal impairment, to which the study authors attributed the lack of response [11]. Available evidence for patients with significant renal impairment, but without significant comorbidities, is presently limited to case reports. These suggest though that the switch to TAF is capable of ameliorating significant TDF-induced renal dysfunction [17, 18], even to the point of reversing Fanconi syndrome [19].

The main limitation of this study was the sample size. Although a highly significant trend between lower pre-switch eGFR and eGFR improvement post-switch to TAF was observed, further study will be needed in larger patient groups to better characterize the relationship between baseline eGFR change and the eGFR response in patients switched from TDF to TAF-containing regimens. There were also no data collected on comorbidities and renal risk factors. These will be the subject of further investigation, but are unlikely to have compromised the validity of our current findings as these have not been found to be associated with TDF-mediated decline in renal function [20]. Finally, improvements in eGFR and proteinuria have been demonstrated in as little as 4 weeks [10] and these changes have been shown to be durable, lasting until at least 96 weeks after switching [21]. As such, the small variability in the time between pre and post-switch laboratory measurements in our study (between 3 and 12 months) is unlikely to have introduced any significant bias to our findings.

TDF remains a recommended first-line antiretroviral agent in major international guidelines [1, 3]. For those patients who go on to experience TDF-induced renal dysfunction, our study suggests that HIV-positive patients with poorer renal function can expect a greater improvement in eGFR upon switching from a TDF- to a TAF-containing antiretroviral regimen.

## Conclusion

Switching HIV-positive patients from TDF to TAF did not lead to an overall change in eGFR. While there was no significant association between age, cumulative TDF exposure and baseline uPCR in such patients, baseline eGFR was a predictor of eGFR change. This suggests that patients with a lower baseline eGFR on TDF would benefit more from the switch to a TAF-containing regimen, but confirmation awaits larger studies.

## Data Availability

The data for our project is retained on the REDCap data system, administered by the Sydney Local Health District. Data availability must be on an “upon reasonable request” basis (made to the corresponding author), to comply with the requirements of the approving human research ethics committee.

## Abbreviations

cART: combination antiretroviral therapy
COBI: cobicistat
E: elvitegravir
EFV: efavirenz
eGFR: estimated glomerular filtration rate
FTC: emtricitabine
RPV: rilpivirine
TAF: tenofovir alafenamide
TDF: tenofovir disoproxil fumarate
uPCR: urinary protein to creatinine ratio
